# Prevention-centered health care in Germany – a nation in need to turn the tide

**DOI:** 10.1007/s10654-023-01030-3

**Published:** 2023-07-31

**Authors:** Stephan Baldus, Karl Lauterbach

**Affiliations:** 1 Department of Cardiology, Heart Center, University Hospital of Cologne, Cologne, Germany; 2 German Ministry of Health, Berlin, Germany; 3 Harvard Chan School of Public Health, Harvard University, Boston, MA USA

Life expectancy is an objective measure of a population´s health and relies on a complex variety of individual, social, economic and regional factors [1–5]. Although the life span with the interruption of World War II has risen globally over the last 100 years, the decline in mortality has recently slowed down in developed countries despite major increases in health expenditure [6–8]. Also, there is robust evidence from European countries that the overall gain in life span appears to be dearly bought by an increase in disabled years [9].

Along these lines, Jasilionis et al. now draws a grim picture on the development of life expectancy in Germany [10]. By means of comparative analyses of life expectancy based on life tables obtained from the Human Mortality Database and subsequent age and cause-specific decomposition, the authors provide sound evidence that Germany currently underperforms in gaining life expectancy as compared to other developed countries. This clear underperformance, in particular in the older population was observed despite Germany´s well resourced health care and welfare systems, its stable economic growth, the low poverty and crime rates and the country’s easy to access infrastructure. Of note, this excess in mortality compared to other countries appears mainly to be driven by cardiovascular mortality, which – as indicated by the authors - is in large part attributive to an underperformance of primary care and disease prevention. Thus, this paper complements recently published data also pointing towards an attenuated increase in expected lifetime gain in Germany as compared to other developed countries [6].

Despite all pharmacological, interventional and preventive effort, cardiovascular disease (CVD) remains the leading cause of death worldwide [11]. Over the last 40 years we witnessed remarkable achievements in the treatment of CVD which translated into a significant decline in cardiovascular mortality: Therapies like acute transcatheter treatment of coronary occlusion in acute myocardial infarction, the implantation of defibrillators in patients at risk for malignant arrhythmias or the advent of novel pharmacological treatments for heart failure drove the decline in cardiovascular mortality by up to 70% over the past 40 years [12]. The invention and use of statins from the middle of the 90ies of the last century also had a major impact on coronary artery disease. Yet, the global disease burden remains high and is still on the rise [11]. This is in large part driven by a critical increase of prevalent CVD in an aging population [5, 11]: Although the age-standardized prevalence of CVD in Germany has declined from 1990 to 2015, the absolute prevalence of CVD has increased by more than 1,5 million cases in 2015 as compared to 1990 [13].

Classical risk factors of CVD are highly modifiable and include arterial hypertension, hypercholesterinemia, diabetes, smoking and dietary habits [14]. They attribute to nearly 70% of all cardiovascular disease in high income countries [15]. The prevalence of these risk factors in Germany is above the Organisation for Economic Co-operation and Development (OECD) average [16]. While Germany faces a large body of inhabitants at risk for cardiovascular disease, only a minority is diagnosed and a fraction receives adequate therapy: For example, less than 50% of Germans with dyslipidemia have been identified as diseased and only 30% receive lipid-lowering medication [17]. Diagnosis of arterial hypertension in young males between 18 and 29 years is missed in up to 76% [18]. And given that the number of active smokers among those between the age of 18–79 remains 30%, the prevalence of CVD is projected to increase further [17–20]. This is clearly not acceptible.

How can Germany learn from other developed countries in tackling cardiovascular morbidity and mortality? Primary prevention of CVD will remain a cornerstone in this regard, which requires systematic identification and vivid control of cardiovascular risk factors. Japan, a well performing developed country implemented legislation and a nationwide strategy specifically promoting primary prevention. This includes guidelines for population-wide health checks, a national Health and Nutrition Survey and incentives for healthy behavior. Additionally, Japan has a strong tradition of providing healthy food in schools; and education on food and nutrition is officially part of the scholar´s curriculum [21]. These measures most likely attribute to the significantly lower prevalence of cardiovascular risk factors and CVD in Japan as compared to Germany [22]. Additionally, optimal secondary prevention of cardiovascular risk factors will be required to improve primary prevention of CVD due to the high prevalence of these risk factors in Germany. A cornerstone for improved treatment of risk factors is the identification of genetic carriers of disease: For exmaple, the Dutch screening program for familial hypercholesterolemia, a disease with an overall prevalence of approximately 0,4%, proved efficient in this regard [23, 24].

These examples demonstrate that it should be possible to improve cardiovascular health in a developed country and call for a nationwide initiative targeting CV prevention and early treatment of cardiovascular diseases in Germany. In fact, the recently founded National Heart Alliance, a confederation of all German cardiovascular associations and societies under the patronage of the German Ministry of Health has now proposed similar projects aiming to reduce cardiovascular disease burden in this country [25].

The National Heart Alliance not only aims at early detection of risk factors for CV disease, it also tries to improve nationwide data collection and accessibility: Denmark has seen remarkable improvement in the treatment of cardiovascular risk factors upon implementation of an electronic health record system with automated data collection from primary care givers [26]. And Sweden implemented several high quality registries tracking disease entities firmly linked to national disease burden such as the Swedish Web-system for Enhancement and Development of Evidence-based care in Heart disease Evaluated According to Recommended Therapies (SWEDEHEART) [27].

Although Germany has recently introduced Electronic Health Records, technical challenges and concerns on data protection still make its implementation challenging [28]. As of today, Electronic Health Records are used by less than 1% of German health insurance policy holders, which calls for action by both the legislation as well as health care providers. Germany will pass legislation for an opt out version of its electronic health record in the fall of 2023 and aims at a 80% coverage of its population by fall 2025.

Although the data provided by Jasilionis et al. for Germany are alarming, there is also light for encouragement: Germany possesses a highly developed health care system which is easy to access, the country is known for its macroeconomic performance. Thus, a powerful and nationwide initiative on primary prevention should be achievable. Such a program would have the potential to be highly efficient both in terms of reducing mortality and also health care costs: [29, 30] We know that the number needed to screen to prevent one death is roughly 10 times lower when screening for dyslipidemia or hypertension as compared to programs tackling cancer or hepatitis, respectively - and thus highly cost effective [31, 32] Therefore, improving primary prevention for CVD might ultimately also cut health care expenditures.

In summary, lowering cardiovascular mortality in Germany is urgently needed and will require combined efforts of primary and tertiary care givers, researchers, and politicians. There is need to embrace initiatives helping to change nutritional behavior on a public and individual level, to implement powerful screening programs, to improve guideline adherence in primary care and to develop novel treatment strategies based on digitalization and adequate allocation of research funds. A more aggressive approach against tobacco use will also have to be considered. This will ultimately help to not only increase the overall numerical life expectancy but increase the number of years free of disability – the most important goal of prevention and early detection of chronic diseases. In the end, Germany could make its way from Saulus to Paulus of cardiovascular disease prevention and evolve as a role model for a modern nationwide prevention-centered health care plan with a focus on cardiovascular disease.
